# Testing a Structural Equation Model of Nursing Home Organizational Characteristics

**DOI:** 10.1093/geroni/igaf122.3854

**Published:** 2025-12-31

**Authors:** Sorah Levy, Sarah Holmes, Barbara Resnick, Kelly Doran, Elizabeth Galik, Tara McMullen

**Affiliations:** University of Maryland, Baltimore, Maryland, United States; University of Maryland School of Nursing, Baltimore, Maryland, United States; University of Maryland, Baltimore, Maryland, United States; University of Maryland, School of Nursing, Baltimore, Maryland, United States; University of Maryland School of Nursing, Baltimore, Maryland, United States; Centers for Medicare & Medicaid Services, Centers for Medicare and Medicaid Services, Maryland, United States

## Abstract

Nursing home (NH) organizational characteristics such as staffing, profit status, and community size are important contributors to the quality of care delivered. These organizational characteristics are linked to resident outcomes such as the prevalence of pressure injuries, rehospitalization, morbidity, and mortality. Guided by the Social Ecological Model and Donabedian’s Structure-Process-Outcome framework, this study tested a comprehensive measurement model of NH organizational factors. The hypothesized measurement model specified two latent factors: Operational (profit status, size, percent Medicaid) and Care Delivery (minutes per resident per day RN, LPN, CNA). This cross-sectional secondary data analysis included 55 NH communities in Maryland and Pennsylvania using data from the Evidence Integration Triangle for Behavioral and Psychological Symptoms of Dementia (EIT-4-BPSD) combined with data from LTCFocus. Structural equation modeling using Mplus was used to test the model. Fit statistics include χ² /df, Tucker Lewis Index (TLI), and root mean square error of approximation (RMSEA). The model demonstrated excellent fit (χ² /df = 0.798, TLI = 1.00, RMSEA = 0.000). CNA staffing was a significant indicator for the Care Delivery factor (β = 0.565, p = 0.008), and percent Medicaid (β = 0.950, p < 0.001) and profit status (β = 0.485, p < 0.001) were significant indicators of the Operational factor. This study provides empirical support for a theoretically based measurement model of NH organizational factors. Future research should explore the impact of NH organizational factors on resident outcomes. Findings may also inform policy and operating decisions aimed at strengthening infrastructure to support quality care.

